# The Effect of Multi-Parametric Magnetic Resonance Imaging in Standard of Care for Nonalcoholic Fatty Liver Disease: Protocol for a Randomized Control Trial

**DOI:** 10.2196/19189

**Published:** 2020-10-26

**Authors:** Dimitar Tonev, Elizabeth Shumbayawonda, Louise Ann Tetlow, Laura Herdman, Marika French, Soubera Rymell, Helena Thomaides-Brears, Filipe Caseiro-Alves, Miguel Castelo-Branco, Carlos Ferreira, Minneke Coenraad, Hildo Lamb, Meinrad Beer, Matt Kelly, Rajarshi Banerjee, Matthias Dollinger, RADIcAL1

**Affiliations:** 1 Perspectum Ltd Oxford United Kingdom; 2 University of Coimbra Coimbra Portugal; 3 Leiden University Leiden Netherlands; 4 University Klinik Ulm Ulm Germany; 5 See Authors' Contributions

**Keywords:** NAFLD, NASH, multiparametric MRI, health economics, biomarker

## Abstract

**Background:**

The rising prevalence of nonalcoholic fatty liver disease (NAFLD) and the more aggressive subtype, nonalcoholic steatohepatitis (NASH), is a global public health concern. Left untreated, NAFLD/NASH can lead to cirrhosis, liver failure, and death. The current standard for diagnosing and staging liver disease is a liver biopsy, which is costly, invasive, and carries risk for the patient. Therefore, there is a growing need for a reliable, feasible, and cost-effective, noninvasive diagnostic tool for these conditions. LiverMultiScan is one such promising tool that uses multi-parametric magnetic resonance imaging (mpMRI) to characterize liver tissue and to aid in the diagnosis and monitoring of liver diseases of various etiologies.

**Objective:**

The primary objective of this trial (RADIcAL1) is to evaluate the cost-effectiveness of the introduction of LiverMultiScan as a standardized diagnostic test for liver disease in comparison to standard care for NAFLD, in different EU territories.

**Methods:**

RADIcAL1 is a multi-center randomized control trial with 2 arms conducted in 4 European territories (13 sites, from across Germany, Netherlands, Portugal, and the United Kingdom). In total, 1072 adult patients with suspected fatty liver disease will be randomized to be treated according to the result of the mpMRI in the intervention arm, so that further diagnostic evaluation is recommended only when values for metrics of liver fat or fibro-inflammation are elevated. Patients in the control arm will be treated as per center guidelines for standard of care. The primary outcome for this trial is to compare the difference in the proportion of patients with suspected NAFLD incurring liver-related hospital consultations or liver biopsies between the study arms, from the date of randomization to the end of the study follow-up. Secondary outcomes include patient feedback from a patient satisfaction questionnaire, at baseline and all follow-up visits to the end of the study, and time, from randomization to diagnosis by the physician, as recorded at the final follow-up visit.

**Results:**

This trial is currently open for recruitment. The anticipated completion date for the study is December 2020.

**Conclusions:**

This randomized controlled trial will provide the evidence to accelerate decision making regarding the inclusion of mpMRI-based tools in existing NAFLD/NASH clinical care. RADIcAL1 is among the first and largest European health economic studies of imaging technologies for fatty liver disease. Strengths of the trial include a high-quality research design and an in-depth assessment of the implementation of the cost-effectiveness of the mpMRI diagnostic. If effective, the trial may highlight the health economic burden on tertiary-referral hepatology clinics imposed by unnecessary consultations and invasive diagnostic investigations, and demonstrate that including LiverMultiScan as a NAFLD diagnostic test may be cost-effective compared to liver-related hospital consultations or liver biopsies.

**Trial Registration:**

ClinicalTrials.gov NCT03289897 https://clinicaltrials.gov/ct2/show/NCT03289897

**International Registered Report Identifier (IRRID):**

DERR1-10.2196/19189

## Introduction

Nonalcoholic fatty liver disease (NAFLD) is the most common cause of abnormal liver blood tests with an estimated prevalence of between 20%-30% in Europe, and higher in the United States [1,2]. The condition is associated with obesity, insulin resistance, and heart disease, resulting in patients with fatty liver disease being twice as likely to get early coronary artery disease compared to the healthy population [3,4]. If left untreated, NAFLD can progress to nonalcoholic steatohepatitis (NASH), characterized by tissue scarring steatosis, lobular inflammation, fibrosis, and ballooning, before the ultimate development of cirrhosis and liver failure. Due to the steady increase of NALFD over the years, NASH has now become the leading cause of liver failure in the developed world, with reported predictions that it will become the leading cause of liver transplant over the coming decades [5]. NASH is a major public health concern and, with a global prevalence of 1.5%-6.5% [6,7] (and 12% in Western populations [8]), poses a significant economic burden on health care institutions.

Similar to most liver diseases, the diagnostic gold standard for NAFLD/NASH is percutaneous liver biopsy [5,9,10]. However, biopsies are painful and carry risk, as 1 out of 1000 people experience serious adverse events, including bleeding, infection, and bowel perforation [11-13]. Biopsies only sample a small part of the liver (approximately 1/50000^th^ of the liver volume) [14] and suffer from sampling location variability, thus affecting the reported stage of fibrosis in up to 50% of cases [15,16], as well as inter-reader variability, which can result in biopsy-finding disagreements amongst pathologists [17-19]. Thus, biopsies alone are not enough to obtain a diagnosis or to monitor liver disease [20,21]. In addition, with the advance of various pathophysiological changes exhibited through liver disease etiologies, some patients experience impaired clotting of their blood due to liver dysfunction [22] and are consequently at a higher risk of experiencing a combination of the risk factors associated with biopsy [8]. The resulting longer hospital stays and increased socioeconomic burden after the procedure make biopsy an unpopular option amongst patients, clinicians, and payers [9]. Although recommended by clinical guidelines as the gold standard for diagnosis and monitoring [10], in practice, liver biopsies are not routinely used unless the patient presents with moderate to severe liver disease or when there is a need to exclude other liver diseases such as autoimmune hepatitis [23]. In light of these factors, health institutions deviate from diagnostic pathways to stratify the risk of advanced liver disease and to postpone, or even replace, biopsy within this population, which has resulted in a nonstandardized care pathway. Therefore, in the absence of biopsy and a universal ground truth in routine care, there is a clear need for noninvasive, objective, discriminatory tests that can stratify normal liver, simple steatosis, steatohepatitis, and cirrhosis. These tests can then be used as a common reference point for clinical care.

Over the years, various noninvasive techniques such as ultrasound, transient elastography (FibroScan; Echosens), diffuse-weighted imaging, magnetic resonance elastography, T1 mapping, and multi-parametric magnetic resonance imaging (mpMRI) have been developed for use as surrogate markers to both diagnose and monitor NAFLD/NASH disease alongside blood tests [24,25]. mpMRI (Liver*MultiScan*; Perspectum Ltd) is an emerging quantitative mpMRI test, the first to combine corrected T1, proton density fat fraction (PDFF), and T2-star, and which can identify the early stages of liver disease [26,27] and predict clinical outcomes accurately [8]. mpMRI also has the potential to become a standardized, consistent step along the NASH clinical diagnostic pathway in multiple health care systems across Europe, as it is cost-saving, noninvasive, fast, repeatable, reliable, and standardized across multiple magnetic resonance vendors [8,9,27-30].

The cost benefits of introducing a noninvasive diagnostic test that detects earlier stage disease may be especially beneficial in the clinical care of people suspected of having fatty liver disease or diabetes [27,31]. The absence of a clear consensus over patient clinical management for suspected fatty liver disease [9,29] necessitates an assessment of mpMRI within existing health care systems to identify potential real-world cost-effectiveness of new imaging technologies and streamline health care for patients. Thus, to investigate the utility and cost-benefit of adding mpMRI into the care pathway of those with suspected NAFLD in Europe (European Union territories and United Kingdom [UK]), this randomized, multi-center, phase 4 control trial to investigate the use of mpMRI as a standardized diagnostic test for NAFLD/NASH was designed. With up to 13 sites across Europe included in this trial, the primary outcome is to compare between the study arms the difference in proportion of patients with suspected NAFLD incurring liver-related hospital consultations or liver biopsies, from the date of randomization to the end of the study follow-up. This will highlight the health economic burden on tertiary-referral hepatology clinics imposed by unnecessary additional consultations.

## Methods

### The Study

RADIcAL1 is a multi-center, phase 4, randomized controlled trial (NCT03289897) which aims to recruit 1072 patients from 13 sites in 4 different European territories, namely Ulm (Germany), Leiden (Netherlands), Coimbra (Portugal), and the UK (Liverpool, Southampton, Dundee, Glasgow, London, Manchester). The 5-year study consists of a 1-year study setup, a 3-year recruitment phase, and an up-to-12-months follow-up. The protocol, informed consent form, participant information sheet, and any proposed advertising material was submitted to each host institution’s appropriate research ethics committee for written approval; a favorable (and granted) response was received in Ulm (198/17), Leiden (P17.076), Coimbra (CE-030/2017), and UK (18/SC/0725).

### Patient Randomization and Study Participants

**Figure 1:**
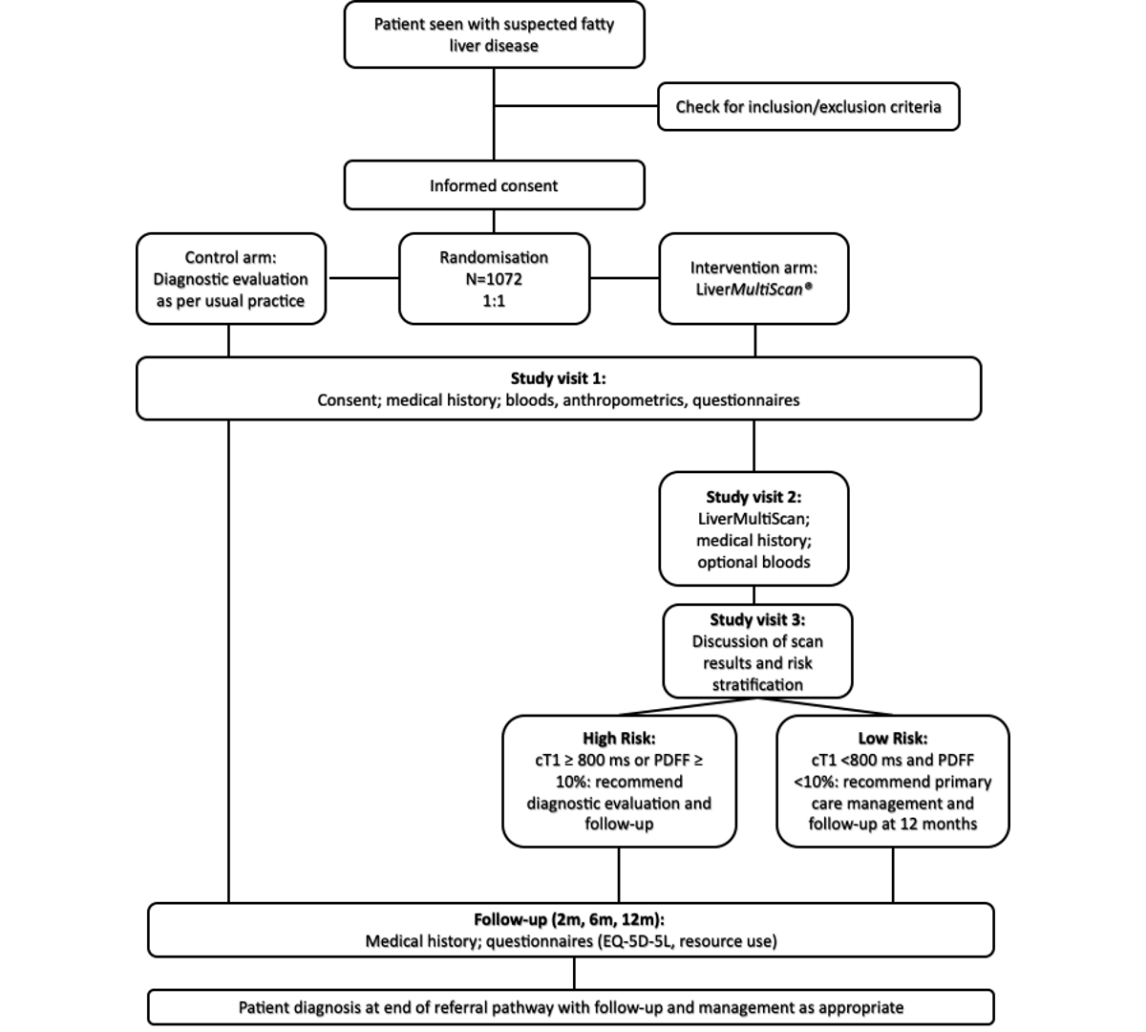
Summary of study visits for participants in the RADIcAL1 clinical trial. m: months; PDFF: proton density fat fraction.

Patients will be randomized using a 1:1 allocation, without blinding, into an intervention arm (with mpMRI intervention) and a control arm (Figure 1). Randomization is automatically calculated using a random number generator on patients that have been already stratified based on a combination of the inclusion criteria (Textbox 1) and the recruitment site. Patients in the control arm will be treated as per center standard of care, with patients following local practice for NAFLD to potentially include physician consultations and anthropometric blood, imaging, and histological assessments [32-34].

**Inclusion and exclusion criteria used during recruitment in the RADIcAL1 trial. NAFLD: nonalcoholic fatty liver disease.**

**Inclusion Criteria**

- Male and female patients, aged 18-75 years, due to undergo an evaluation for suspected NAFLD
- Within standard of care, presence of either (1) elevated liver function tests (ALT, AST, or GGT ≥1.5 x upper limit of normal, and ALT, AST ≤5 x upper limit of normal) up to 1 year prior to patient recruitment, OR (2) imaging suggestive of fatty liver disease up to 3 years prior to patient recruitment
- OR presence of ≥ 3 of the following criteria: (1) insulin resistance or type 2 diabetes mellitus, (2) obesity (BMI >30 or waist-to-hip ratio > 1.00 for men / >0.85 for women), (3) hypertension (≥130/85 mmHg), (4) elevated triglycerides (≥1.7 mmol/l), (5) low HDL-cholesterol (<1.05 mmol/l for men / <1.25 mmol/l for women)
- Participant is willing and able to give informed consent for participation in the study

**Exclusion Criteria**

- Participants may not enter the study if they have any contraindication to magnetic resonance imaging (including pregnancy, extensive tattoos, pacemaker, shrapnel injury, severe claustrophobia)
- Patients with proven liver disease other than NAFLD
- Liver transplantation
- Patients that present with clinical signs of chronic liver failure (variceal bleeding, ascites, overt encephalopathy)
- Alcohol overuse/abuse as determined by local guidelines
- Patient with known malignant liver tumors and those with any malignancy with life expectancy <36 months
- Heart failure (New York Heart Association: stages II-IV)
- Severe mental illness
- Any other cause, including a significant disease or disorder which, in the opinion of the investigator, may either put the participant at risk because of participation in the study or may influence the result of the study, or the participant’s ability to participate in the study

Those in the intervention arm will be treated according to the result of the mpMRI scan; if the liver fat is ≥10% or fibro-inflammation is identified (corrected T1 (cT1)≥800 ms), then further diagnostic evaluation will be recommended (such as further monitoring of liver enzymes, repeat mpMRI assessment at 6-12 months, assessment of liver stiffness, or assessment of response to lifestyle management activities) [10,35]. Otherwise, management in primary care for 12 months will be recommended. In both arms, clinical choices will be patient and site-specific in adherence with NAFLD guideline recommendations [32-34].

Potential participants will be recruited from (1) general practitioners or specialists from tertiary care hospital consultations (eg, obesity consultation); (2) secondary care clinics, and (3) databases from previous ethically approved studies where patients have consented to have their contact details retained in order to be contacted if eligible to take part in other studies.

During recruitment, the inclusion and exclusion criteria highlighted in Textbox 1 will be used to identify potential participants.

Once a potential participant expresses interest in the study, they will be provided with a patient information leaflet for a minimum of 24 hours and an opportunity to discuss their eligibility and the details of the study. In accordance with good clinical practice, the participant is free to withdraw from the study at any time for any reason without prejudice to current or future care. For those participants who wish to withdraw from the study, the option to permit ongoing use of data and samples which have already been collected, as well as future recording and usage of routinely collected clinical data and results, will be given. This will be clearly documented in the patient consent form. In addition to this, patients who are unable to undergo the magnetic resonance imaging (MRI) scan (eg, due to claustrophobia) may also be withdrawn from the study.

### Study Visits

Patients will be required to attend their respective clinical centers for up to 3 dedicated study visits, as summarized in Figure 1. At Visit 1, informed consent, medical history, anthropometric readings, and blood will be taken. Visit 2 is for the intervention arm only; patients will be required to fast for 4 hours before the Liver*MultiScan* MRI scan (standardized imaging protocol in a 1.5 or 3T MRI scanner following the Perspectum protocol), which will involve lying supine in the MRI scanner for 10-15 minutes. At this visit, optional blood samples for further tests may be taken. Visit 3 is for the intervention arm only, during which clinicians will discuss the results of the MRI scan with the patients and change patient management if appropriate.

Once they have had their scan, patients will be followed up for a period of 6-12 months. In this trial, patients will also be requested to complete questionnaires at recruitment 2, 6, and 12 months after entering the study. In this trial, patients will also be requested to complete a resource use and quality of life (EQ-5D-5L [36]) questionnaire at recruitment and 2, 6, and 12 months after entering the study. Those in the intervention arm will also be asked to complete an MRI satisfaction questionnaire after having their mpMRI.

The resource use questionnaires completed at randomization will cover information such as appointments that the participant has had with a health care professional (inpatient and outpatient), medication usage, diet and physical exercise, paid and unpaid help the participant may require, and their insurance coverage. Furthermore, at the 2-, 6-, and 12-month follow-up visits, participants will be asked to answer additional questions regarding changes in medication and medical examinations they received as an outpatient. These examinations include blood tests, ultrasounds, other imaging (eg, endoscopy, CT scan, and MRI), and biopsies.

The EQ-5D-5L questionnaire [36] asks participants to describe their health on the day of questionnaire completion in order to assess the impact on quality of life. Each participant must rate their mobility, self-care, usual activities, pain or discomfort, and anxiety or depression. The participant has 5 options to pick from for each parameter or question: no problem, slight problem, moderate problem, severe problem, or unable to carry out the task. Finally, participants are asked to indicate how they think their health is on that day, on a scale of 0 to 100.

### Intervention

**Figure 2:**
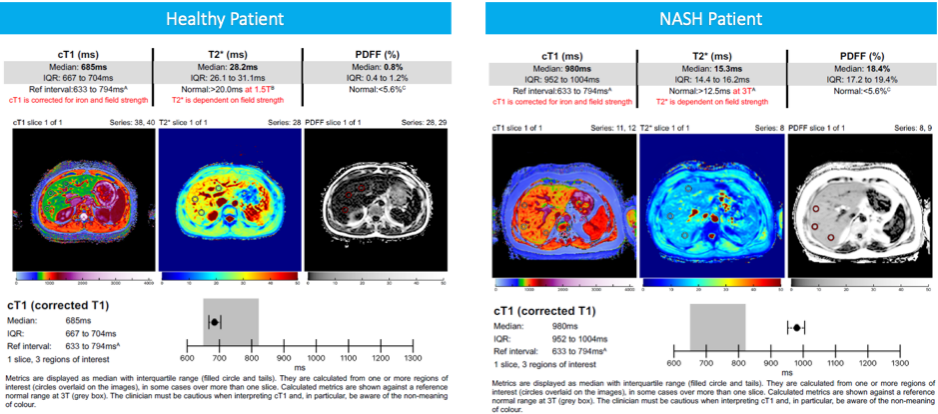
Sample reports that demonstrate the information that can be derived from the use of mpMRI to aid as a diagnostic tool for clinicians, highlighting the differences between a healthy patient with low cT1 and a patient with suspected nonalcoholic fatty liver disease (NAFLD) and high cT1. PDFF: proton density fat fraction.

MRI-derived biomarkers provide many opportunities for diagnostic enrichment. MRI exploits the magnetic properties of hydrogen nuclei protons within a determined magnetic field. T1 mapping measures longitudinal relaxation time and, thus, is a surrogate measure of the amount of water present or the structural distribution of water molecules (ie, T1 can be used to indicate whether tissue water is freely moving, is structured within cells, or is bound to macromolecules). Therefore, as T1 relaxation time lengthens with increases in extracellular fluid, it has shown promise as an effective biomarker of inflammation and fibrosis in several organs [37,38]. The presence of iron in the liver, however, which can be accurately measured from MRI-T2-star relaxation time, shortens the T1 and thus must be accounted for [39]. An algorithm has been created by Perspectum Ltd that allows for the bias introduced by elevated iron to be removed from the T1 measurements, yielding the iron-corrected T1 (cT1) [14,39]. MRI-PDFF is a ratio expressed as a percentage of the fraction of the MRI-visible protons attributable to fat divided by all MRI-visible protons in that region of the liver attributable to fat and water. Taking advantage of the chemical shift between fat and water, pulse sequences (including fast spin-echo and gradient-recalled echo sequences) can be used to acquire images at multiple echo times at which fat and water signals have different phases relative to each other. cT1 maps have been shown to be correlated with fibro-inflammation and predictive of clinical events [8,14]. PDFF has been shown to have an excellent correlation with histologically graded steatosis across the clinical range seen in NASH and high diagnostic accuracy in stratification of all grades of liver steatosis. Hence, together, PDFF and cT1 hold promise to accurately assess all relevant aspects of liver disease: fat, inflammation, and fibrosis [25,40]. The sample reports shown in Figure 2 demonstrate the information that can be derived from the use of mpMRI to aid as a diagnostic tool for clinicians, highlighting the differences between a healthy patient with low cT1 and a patient with suspected NAFLD and high cT1. The high repeatability and reproducibility of mpMRI (both coefficients of variation equalling 3.3% for cT1 [25,28,41]) and predefined diagnostic thresholds for an mpMRI recommendation make clinical misinterpretation of the mpMRI unlikely.

### Objectives and Outcomes

All primary and secondary objectives and outcome measures are outlined in Table 1. The primary objective of the study is to compare the cost-effectiveness of the standard of care received by patients with suspected NAFLD in the stated EU territories compared to the care such patients will receive when Liver*MultiScan* is introduced as a standardized diagnostic test. The primary endpoint of the study utilizes health care resource use data to compare the difference in proportion of patients incurring liver-related hospital consultations or liver biopsies, from the date of randomization to the end of the study follow-up, with cost-effectiveness dependent on local jurisdiction. One secondary objective will be based on data from patient satisfaction questionnaires to explore the implementation of the intervention. Another secondary objective investigating the certainty and frequency of diagnosis is based on clinician response to a specific question (“Using all the information obtained to date, how certain are you to make a diagnosis of NAFLD today?”) posed at each diagnostic visit in the patient’s journey. The 4 possible predefined responses are further subgrouped into binary categories, one subgroup for certainty and another for frequency of diagnosis. Other secondary objectives include a comparison of the time to diagnosis, which utilizes data based on any liver-related diagnosis [from 7 options: primary nonalcoholic fatty liver (NAFL), secondary NAFL, primary NASH, secondary NASH, mixed-etiology NAFL, mixed-etiology NASH, other etiology]. Additionally, the use of resources, the actual costs over a 12-month period, and the level of skill or clinical specialization required within each study arm will be investigated as secondary objectives, based on the resource use questionnaires and study case report forms. Exploratory objectives include a model looking at long-term cost-effectiveness based on quality of life over a lifetime horizon, using the EQ-5D-5L data and an analysis of the diagnostic accuracy of mpMRI and other study biomarkers.

**Table 1:** RADIcAL1 primary and secondary objectives.

Criteria	Primary	Secondary
**Objectives**	To investigate whether the introduction of mpMRI as a standardized diagnostic test for liver disease can prove a cost-effective method in different European territories	To investigate patient satisfaction with mpMRI instead of existing care (with other liver investigations)
		To investigate the certainty and frequency of diagnosis at points of time in the patient pathway
		To investigate which pathway is quicker to get to the diagnosis as recorded at the final follow-up visit (including all corrections and additional investigations)
		To measure which health care resources and costs are required in the 2 diagnostic pathways
		To investigate the cost-effectiveness of mpMRI against standard of care
		To investigate skills/specialization required
**Outcome measures/ endpoints**	The proportion of patients with suspected NAFLD incurring liver-related hospital consultations and/or liver biopsies, from the date of randomization to the end of the study follow-up	Patient feedback from patient satisfaction questionnaire, at baseline and all follow-up visits to the end of the study
		Certainty of diagnosis is defined as a binary (*yes/no*, as opposed to *unlikely/probable*) and frequency (*yes/probable*, as opposed to *no/unlikely*), at baseline and all follow-up visits to the end of the study
		Time, from randomization to diagnosis by the physician, as recorded at the final follow-up visit
		Rates of liver-related outpatient investigations/ consultations/hospital admissions per 400 patients during the study
		Cost of mpMRI based on randomized comparison
		Personnel required to perform procedures and tasks, from the date of randomization to the end of the study follow-up

### Sample Size Calculation

In a study by Blake et al [9], it was identified that the use of Liver*MultiScan* can result in a decrease in biopsy of 18%. Adopting a conservative target of identifying a 14% decrease across different regions, to maintain statistical significance [with more than 80% power (α=.05) to show a difference in proportion of patients having consultations between the 2 pathways), each randomization arm is required to have 402 patients. Moreover, due to the size of the trial, the final recruitment target was powered to include a 25% dropout rate (including those lost to follow-up during the completion of the study). Thus, a total cohort of 1072 patients with suspected fatty liver disease will need to be recruited into the trial.

### Statistical Methods and Data Management Plan

Statistical support for all primary and secondary analyses will be provided by Perspectum Ltd. Detailed health economic and statistical analysis plans [42] (Multimedia Appendix 1) describe the required analyses to investigate the study objectives. These include details of standard statistical analyses [*t* test, analysis of variance, area under the receiver operating curve (AUROC)] and data analysis packages [such as R (R Core Team), MATLAB (MathWorks), and Python (Python Software Foundation)], which will be used to report summary statistics for patients in both arms of the study. Moreover, summary statistics will be reported (number of observations, mean, standard deviation, or percentages, as relevant) for the demographic variables, clinical variables, and outcomes for the total group, and comparisons with any noninvasive tests offered in their care.

The health economic analysis will evaluate changes in resource use and costs for data collected from randomization to the end of the study. Within this evaluation, the determination of the cost-effectiveness of mpMRI from the perspective of each health care system following the intention-to-treat principle will be derived. Health care resource use (including diagnostic procedures, health care consultations, and hospital admissions) will be obtained from medical records as well as via patient self-reported data during follow-up visits. A detailed health economic analysis plan, detailing the methods used and models developed using study data, will also be agreed upon and developed prior to the end of the study (Carolan et al., unpublished data).

Additional exploratory analysis will evaluate the diagnostic performance of cT1 and PDFF using AUROC, and will assess the concurrence of mpMRI metrics with other surrogate biomarkers associated with NAFLD/NASH used more regularly in clinical practice, such as glucose and hemoglobin A1C (HbA1c, a measure of glycated hemoglobin which contributes to diabetes diagnosis), enhanced liver fibrosis tests (used to test for advanced liver fibrosis in patients with NALFD), and cholesterol, utilizing correlation analyses (Pearson correlation for normally distributed data, and Spearman rho for non-normally distributed data). The concordance of mpMRI metrics and biopsy data will be assessed using Cohen kappa (κ) statistic, Bland-Altman analysis (bias, limits of agreement, and the corresponding 95% confidence interval), Pearson correlation, and mean coefficient of variation, which will be estimated.

In this trial, all data collected will be documented in electronic case report forms. In addition to this, all patient-related data will be handled and stored according to the European and national data protection laws [42]. All outcome data will be analyzed using an intention-to-treat principle, where data from participants shall be analyzed according to the group in which they were randomized, even if they did not receive the allocated intervention.

## Results

RADIcAL1 has been funded from May 2016, and ethics approval was granted in April 2017 (Portugal), July 2017 (Germany, Netherlands), and June 2018 (UK). Data collection began in September 2017 and is estimated to be complete by the end of December 2020. As of April 2020, a total of 726 patients with suspected NAFLD or metabolic syndrome or both have been enrolled. Results will be analyzed by the end of the study, and publication of the results is expected by March 2021.

## Discussion

Nonalcoholic fatty liver disease (NAFLD) and its more progressive form, nonalcoholic steatohepatitis (NASH), are emerging as the most important cause of liver disease worldwide, thought to potentially become the number 1 cause of end-stage liver disease [5]. Their increasing prevalence also share demographic and epidemiological parallels with the worldwide epidemic of obesity and type 2 diabetes mellitus [8,43,44], and the presence of these comorbidities are thought to further increase the risk of cardiovascular disease [44,45]. Due to the increased numbers of patients now requiring both diagnosis and regular monitoring for NAFLD/NASH, great economic and time-related burdens are now being placed upon already strained health care systems [5,44]. Current clinical guidelines and care pathways require patients to undergo liver biopsy for the diagnosis and monitoring of NAFLD/NASH, which is risky, painful, and costly, leading to a reluctance from both patients and clinicians to engage in such procedures with regularity [9], thus highlighting an increasingly urgent requirement for a cost-effective, repeatable, reproducible, and noninvasive tool to aid the diagnostic pathway [41].

To the best of our knowledge, this will be the first large-scale, multi-center study to evaluate the cost-effectiveness of mpMRI within the diagnostic pathway for patients with NAFLD/NASH across multiple European territories. The primary objective of the RADIcAL1 trial is to evaluate the cost-effectiveness of mpMRI within tertiary care units within Europe, assessing the impact of its utility upon the number of unnecessary consultations and biopsies that patients must attend, and the economic burden faced by health care systems. From these findings, RADIcAL1 has the potential to produce concordance and optimization of the diagnosis and monitoring pathways for patients whom, with better knowledge of their NAFLD/NASH status, may be able to undertake informed lifestyle changes and prevent further progression of comorbidities, potentially producing further health-economic savings [5,9]. Qualitative data in RADIcAL1, such as the patient satisfaction survey, will provide patient experience insights directly from a population the mpMRI technology is designed to benefit. Furthermore, due to data collection throughout the clinical care pathway, RADIcAL1 also has the potential to assess both the diagnostic accuracy and speed in which care is received in both study arms, adding further evidence to the requirement for a singular, agreed-upon, ideal diagnostic pathway.

mpMRI is well placed to provide accurate monitoring of individual patient responses to drugs in trials and within the care pathway, allowing researchers and clinicians to make informed decisions regarding patient care, with the potential to optimize the allocation of expensive treatments. We expect the introduction of mpMRI into the standard care pathway for patients with NAFLD/NASH to provide health and socioeconomic benefits to patients in addition to cost-savings for health care providers, and this will be evaluated in RADIcAL1.

## Appendix

Multimedia Appendix 1 Statistical analysis plan.

Multimedia Appendix 2 Grant Protocol Peer Review.

## Abbreviations

AUROC: area under the receiver operating curve
cT1: corrected T1
mpMRI: multi-parametric magnetic resonance imaging
NAFLD: nonalcoholic fatty liver disease
NASH: nonalcoholic steatohepatitis
PDFF: proton density fat fraction
