# Supplementary material for: The Effect of Multi-Parametric Magnetic Resonance Imaging in Standard of Care for Nonalcoholic Fatty Liver Disease: Protocol for a Randomized Control Trial
Source: JMIR Res Protoc. 2020 Oct 26;9(10):e19189. doi: 10.2196/19189 (PMC7652684; doi:10.2196/19189)
Supplement: Multimedia Appendix 1 [file resprot_v9i10e19189_app1.pdf]

Perspectum

Title:	RADIcAL 1 Statistical Analysis Plan
Protocol Version Number:	Version 4.0
Protocol Reference	EC-34
Sponsor	Perspectum
Ethics Reference	18/SC/0275
Trial Registration	NCT03289897
Date of protocol	29th July 2019
Chief Investigator	Professor Matthias Dollinger and Dr Dimitar Tonev

Table of Contents

Approvals *3*

Abbreviations *4*

Introduction *4*

Background and Rationale 4

Objectives 5

Purpose of Statistical Analysis Plan *5*

Study Methods *5*

Trial Design 5

Randomisation 7

Blinding 7

Sample size 7

Statistical Interim Analysis and Stopping Guidance 8

Timing of Final Analysis 8

Timing of Outcome Assessments 8

Statistical Principles *8*

Confidence Values and *P* Values 8

Compliance with visits adherence 8

Analysis Population 8

Trial population *9*

Screening data 9

Eligibility: 9

Recruitment 9

Withdrawal and Follow-up 9

Demographics and 9

Baseline Patient Characteristics 9

Analysis *10*

Outcome Definitions 10

Analyses Methods 10

Missing data 10
Statistical software 11
Reporting Conventions 11
References *11*
Appendix A: RADIcAL 1 Study Objectives *11*
Appendix B: Powering *12*
Appendix C: Timing of Outcome Measures *13*
Appendix D: Reference Ranges *13*
Enrolment 14
Analysis 14
Follow-Up 14

Approvals

Role	Name	Signature
EU Chief Investigator	Matthias Dollinger	M. Dollinger<br>M Dollinger Mar 31, 2020 IP:24.134.253.61
UK Chief Investigator	Dimitar Tonev	D Tonev Mar 26, 20<br>IP:94.1.213.6
Head of Clinical Science	Helena Thomaides-Brears	Helena Thomaides-Brears<br>H Thomaides-Brears Mar 31, 20
Author	Marika French	Marika French<br>M French Mar 31, 2020 IP:62.255.31.130
Biostatistician	Arina Kazimianec	Arina Kazimianec<br>A Kazimianec Mar 31, 202

Validity: Please ensure that the required digital signatures are valid prior to publication of a new issue.

SAP Version Control

Issue	Paragraph	Details	Reason	Date/Initial
0.1	All	New document	First draft for review	13Mar2020 MF
0.2	All	Changes made	Addressed comments after internal review	20Mar2020 MF

Roles and Responsibilities

SAP contributors:	
Author	Arina Kazimianec, MSc, Biostatistician, Marika French, Innovation (both PD)
Advisor	Helena Thomaides-Brears, DPhil, BSc, Head of Clinical Science, PD<br>Andrea Dennis, PhD, Head of Biomarker Science, PD
**Trial Sponsor:**	
Perspectum	Gemini One, 5520 John Smith Drive, Oxford, OX4 2LL, 01865 655343

Roles:	
Funder	Horizon 2020 SME Instrument Programme
Principal Investigator	Professor Matthias Dollinger and Dr Dimitar Tonev
Clinical Project Manager	Laura Herdman

Abbreviations

ALT	Alanine aminotransferase
AST	Aspartate aminotransferase
cT1	Corrected T1
CTU	Clinical Trials Unit
eCRF	Electronic Case Report Form
oGGT	Gamma-glutamyl transferase
GP	General Practitioner
HbA1c	Haemoglobin A1c
HEAP	Health Economic Analysis Plan
LMS	Liver*MultiScan*
LUMC	Leiden University Medical Centre
MAR	Missing at Random
MCAR	Missing Completely at Random
MNAR	Missing Not at Random
MpMRI	Multiparametric Magnetic Resonance Imaging
MRI	Magnetic Resonance Imaging
NAFLD	Non-alcoholic fatty liver disease
NASH	Non-alcoholic steatohepatitis
PDFF	Proton Density Fat Fraction
RCT	Randomised Controlled Trial
SAP	Statistical Analysis Plan
T2DM	Type 2 Diabetes
UKBB	UK BioBank

Introduction

Background and Rationale

RADIcAL-1 investigates the impact and cost-effectiveness of magnetic resonance-derived biomarkers of liver health (cT1, PDFF) in a European cohort with suspected NAFLD and metabolic syndrome. Liver*MultiScan* (LMS) is a medical

device that generates these biomarkers to aid the diagnosis of liver disease (1) and help predict clinical outcomes (2). For further information, please refer to the protocol.

Please refer to the Health Economics Analysis Plan (HEAP) (Deliverable 3.6) for details on the Health Economics analysis and Endpoints/Outcome Measures.

Objectives

The Health Economic Primary Objectives, Secondary Objectives and Endpoints/Outcome Measures as defined in the study protocol can be found in Appendix A: Table 1 . This document outlines the Clinical Objectives, Endpoints/Outcome Measures and analysis methods only.

Clinical Objectives and Outcome Measures

Objective	Endpoint/Outcome Measure
1. Evaluation of the diagnostic performance of cT1 and PDFF for predicting histologically defined disease will be determined where biopsy data is available.	1. The association between mpMRI measures and current disease state evaluated using the biopsy-derived gold standard.
2. Agreement of cT1 and PDFF with other non-invasive diagnostic tools metrics (eg. Fibroscan results).	2. The correlations between mpMRI metrics and other non-invasive surrogate markers of disease.
3. Correlation of cT1 and PDFF with blood derived biomarkers stated in the analysis methods.	3. The correlations between mpMRI metrics with traditional blood serum markers of disease.
4. Investigate whether cT1 and PDFF differ between groups with different suspected disease diagnoses at baseline.	4. Groupwise difference in cT1 and PDFF between subgroups based on suspected disease diagnosis at baseline.
5. Investigate whether cT1 and PDFF differ between groups with different suspected disease diagnoses at 12-month follow-up.	5. Groupwise difference in cT1 and PDFF between subgroups based on final diagnosis, once known.

Purpose of statistical Analysis Plan

The SAP outlines the statistical analysis (methodology and scope) to be undertaken for analysing clinical data collected during the RADIcAL-1 trial. Moreover, it discusses approaches undertaken to ensure the trial has been accurately (re)powered to carry out the outlined analysis.

Study Methods

Trial Design

RADIcAL 1 is a parallel multicentre phase IV randomised control trial to determine the implementation and health care cost of LMS vs routine methodical assessment of NAFLD (control arm) in different EU territories.

At baseline, clinical data is obtained from patients as outlined in the study protocol. Health care resource use is collected at baseline, 2, 6 and 12 months.

Resource use on a per patient basis is being collected in a series of questionnaires throughout the study follow-up period. The questionnaires will be conducted at the baseline visit and then 2 months, 6 months and 12 months post randomisation. Both quantitative data and qualitative data will be collected in the questionnaires. Quantitative data

collected includes the number of secondary outpatient appointments participants incurred and the amount of time and money spent attending appointments and paying for prescriptions. Qualitative data collected includes the types of specialists seen at appointments and comments that participants may have after their MRI scan.

Patients recruited from January 2020 onwards will be limited to a 6 month follow up only (no 12 month follow up) due to the EU Horizon 2020 grant agreement ending on the 31st December 2020, therefore no data will be collected beyond this date.

Participant medical history will be collated once the patient has consented and is enrolled to the study. This data will include any existing comorbidities, blood results and imaging results that may have resulted in the inclusion of a participant. For more detail on the inclusion criteria, please refer to the study protocol.

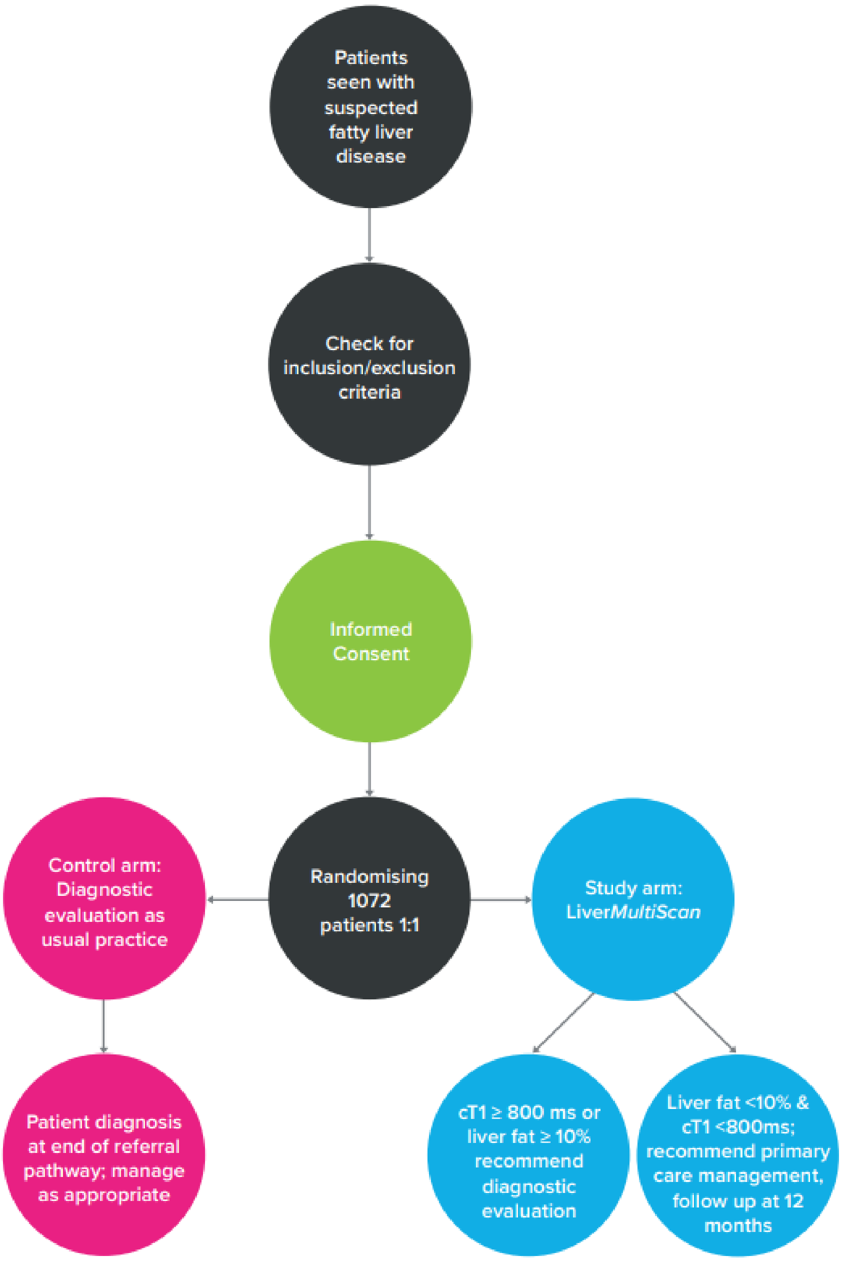

A summary of data that is collected at each time point per arm can be found in Appendix C: Table 1.

Randomisation

In terms of randomisation, there are 72 strata as detailed below:

1. Centre: 1=Ulm, 2=Leiden, 3=Coimbra and 4=UK
2. Sex: 1=Male and 2=Female
3. Age Category: 1=AGE > 17 && AGE < 45, 2=AGE > 44 && AGE < 60 and 3=AGE > 59 && AGE < 76
4. Evidence suggestive for liver disease and/or metabolic syndrome: 1=Both, 2=Only evidence suggestive for liver disease, and 3=Only metabolic syndrome

Blinding

Data has been unblinded for analysis.

Sample size

The sample size for this study was calculated to provide at least 80% power at 5% significant level to detect a 14% reduction in biopsy for patients with suspected NAFLD following the introduction of LMS into the diagnostic pathway. Additionally, this allows for a 25% drop-out rate.

The estimates of NAFLD prevalence, sensitivity and specificity for different imaging tests and expected biopsy rates were based on the literature (3, Table 1). Confusion matrices based on the sensitivity and specificity of Transient Elastography and Multiparametric MRI can found in Appendix B: Table 1 and 2, respectively.

Appendix B: Table 3 uses the imaging test sensitivity and specificity to arrive at a predicted 18% reduction in biopsies following the introduction of Liver*MultiScan*. Sample sizes were also calculated for a more conservative 15% and 14% reduction in biopsies.

<u>Power calculations based on the biopsy reduction rate:</u>

The Power.prop.test (R package "stats") computes the power of the two-sided test for proportions or allows the determination of parameters able to obtain the target power. The required inputs are: N = number of observations per group (sample size per arm), p1=proportion of patients with biopsy in control arm, p2=proportion of patients with biopsy in study arm, significance level=type I error probability, power=1 – type II error probability and alternative=one or two-sided test.

<u>Option 1:</u> 18% reduction in biopsy

Power.prop.test(p1=0.682, p2=0.558, significance level=0.05, power=0.8, alternative= "two sided")

N = 239

<u>Option 2:</u> 15% reduction in biopsy

Power.prop.test(p1=0.682, p2=0.550, significance level=0.05, power=0.8, alternative="two sided")

N = 350

<u>Option 3:</u> 14% reduction in biopsy

Power.prop.test(p1=0.682, p2=0.587, significant level=0.05, power=0.8, alternative="two sided")

N = 402

Using the information above and allowing for a 25% dropout for all powering options above, the total n for each option is:

**Biopsy Reduction (%)**	**18%**	**15%**	**14%**
**N per arm**	239	350	402
**Both arms**	478	700	804
**Expected drop out**	25%	25%	25%
**Total n to recruit**	**637**	**933**	**1072**

The most conservative powering option (3) will be used, therefore the total n will be 1072 with the actual drop-out rate will be recorded in the final study manuscript.

Statistical Interim Analysis and Stopping Guidance

Interim analysis can be defined as data analysis performed while the trial is still ongoing, conducted for ethical reasons (benefit, safety, lack of difference) in addition to investigating the cost, resources and meaningfulness of a project. Possible consequences of an interim analysis would be early stopping for futility or continuation of the study. Interim analysis will take place for use in conference abstract submissions and will include but not be limited to methods highlighted in the Analysis Methods.

In most cases, LMS biomarkers (PDFF and cT1) will be utilised in interim analysis.

The interim analysis will be undertaken by Perspectum. These results will be used to track the progress of the study. In addition, the LUMC CTU will be undertaking an internal analysis.

Timing of Final Analysis

The eCRF (ProMISe) will be locked on 31st December 2020. Statistical analysis to be completed in Q1-Q2 of 2021.

Timing of Outcome Assessments

Patient visits occur between 4th September 2017 and 31st December 2020. The final patient visit will be 31st December 2020, therefore complete clinical data will be available Q1 of 2021. A summary table containing the type of the data to be collected at each time point can be found in Appendix C: Table 1.

Statistical Principles

Confidence Values and *P* Values

Throughout analysis, the significance level of alpha=0.05 and a 95% confidence interval (95%CI) will be utilised.

Compliance with visits adherence

To ensure that participant visits are occurring in line with the protocol, monitoring visits are conducted throughout the study. At these visits, a percentage of participants Case Report Forms, Source Documentation and Questionnaires will be checked. Additionally, quality reports are generated on the eCRF and distributed to the site, highlighting gaps in the database and questions that need to be answered.

Protocol deviations are logged on a site by site basis and a summary will be described in the final study manuscript.

Analysis Population

For the study population please refer to the protocol.

The analysis population will include the full analysis population, those who are randomised into the study and have a complete standard set of data. These participants will have all the necessary baseline data including standard liver blood markers (listed in analysis method 3).

In the context of the statistical analyses to be undertaken, baseline characteristics will be estimated using the full analysis population (all subjects who were randomised with standard set of data).

Diagnostic utility of cT1 and PDFF will be estimated using the protocol population (those randomised into the study arm and have completed at least one MRI resulting in a quantifiable cT1 and PDFF).

Trial population

Screening data

Participants are screened prior to enrolment. Most screen failures are logged on a paper form on-site. A small number of screen failures are logged on ProMISe.

Eligibility:

Refer to protocol

Recruitment

Participants are generally recruited from a range of clinics including hepatology, endocrinology, gastroenterology and NAFLD specific clinics at hospitals in addition to GP surgeries.

Withdrawal and Follow-up

Reasons for withdrawal and lost to follow-up are be reported in the eCRF and will be summarised and tabulated in any publications.

Demographics and Baseline Patient Characteristics

Descriptive statistics will be used to summarise baseline characteristics of the study population for all the patients enrolled in the study regardless of the arm and intervention as well as for each arm separately (and potentially per stratum (strata specified above)). In addition, if a subpopulation of the study cohort is used, for example for the health economic analysis, descriptive statistics will be used to summarise this cohort.

Normality of the data will be determined by use of the Shapiro-Wilk test and visual investigation of histograms. Results will be expressed as mean with standard deviation for continuous normally distributed variables, as median and interquartile range (IQR) for non-normally distributed data, and as counts and/or percentages for categorical variables. Linearity of continuous data will be assessed by scatter plots.

Student's two-sample t-test will be used to compare the means of two independent samples (e.g. treatment arms) for continuous (e.g. cT1, PDFF) normally distributed data (if equal variance assumption is met). Data transformation (e.g. log10) may be performed for the data to conform to a more Normal distribution. Alternatively, Welch's t-test may be performed as it is designed for unequal variances and/or unequal sample sizes (normality assumption must still be met). Analysis of Variance (ANOVA) will be used to test for differences in means between multiple groups for normally distributed data with Tukey's HSD as a post-hoc test. Mann-Whitney U-test will be used for comparison of two groups of continuous data where the data is not normally distributed. Kruskal-Wallis test and Dunn test for multiple comparisons will be used for non-normally distributed data (pairwise Mann-Whitney U-tests with p-values adjusted for

multiple comparisons (e.g. with Benjamini-Hochberg) could be also be used). Chi-squared test will be used for categorical data.

Clinical Laboratory Evaluations

Lab tests performed at more than one timepoint will be summarised via shift tables (baseline vs the latest or the most extreme value). Additionally, scatter plots will be performed with x=baseline value and y = latest value or the most extreme (the dots can also be coloured by treatment arm and the 'order' of the visit may be depicted by a shape (e.g. baseline – round, measurement 3-6 months post-baseline – square, etc).

Analysis

Outcome Definitions

Endpoints/Outcome Measures for RADIcAL 1 can be found in the introduction section.

Analyses Methods

The following analysis is exploratory:

1. Evaluation of the diagnostic performance (i.e., ability to classify disease status) of cT1 and PDFF will be determined using the area under the receiver operative curve (AUROC) based on the sub-cohort of patients where biopsy (i.e., ground truth) data is available. Youden's Index will be computed as well as the corresponding 95% CI of AUROC. Sensitivity and specificity values of each of the biomarkers at representative operating points will be provided.
2. Agreement of measurements of cT1 and PDFF with other non-invasive diagnostic tools metrics (e.g. Fibroscan results) will be assessed using an area under the receiver operative curve (AUROC) based on the sub-cohort of patients where the data is available. Youden's Index will be computed as well as the corresponding 95% CI of AUROC. Sensitivity and specificity values of each of the biomarkers at representative operating points will be provided.
3. Correlation between LMS metrics and blood biomarkers stated in EASL guidelines (4) (including but not limited to AST, ALT, Fasting blood glucose, HbA1C, OGTT, Total blood count, Serum total HDL cholesterol, triacylglycerol and uric acid) using Pearson's correlation for normally distributed data, and Spearman's correlation for non-normally distributed data.
4. Study arm sub analyses: Stratify study arm participants into groups based on the suspected disease diagnosis (NAFLD, Metabolic Syndrome or Both). Using pairwise ANOVA and Tukey's test for post-hoc analysis, the significance between LMS metrics for the study arm cohort and a UKBB control group (defined by BMI<25 and PDFF<5%) will be investigated.
5. Study arm sub analyses: Stratify study arm participants based on the final diagnosis given to the patient (examples include NALFD, NASH, T2DM & Metabolic Syndrome). Using the same analysis methods as method 4, investigate the significance between LMS metrics for the study arm cohort and a UKBB control group (defined above).

Missing data

Missing data can be requested to an extent and will mostly consist of demographics or clinical data flagged in previously mentioned quality reports. These quality reports highlight data missing throughout the entire patient follow-up, including baseline data. The extent/percentage of the missing data (both at baseline and subsequent follow-up) will be quantified for metrics such as demographics, key blood measurements, as well as cT1 and PDFF. Mechanisms of data missingness (MAR, MCAR, MNAR) will be established based on the prior knowledge as well as a number of statistical

(such as correlation analysis to establish dependence of missing data other variables or Little's MCAR test) and graphical approaches (e.g. using VIM package in R which allows visual inspection of missing data via barplots, missing data pattern plots, scatterplots and spineplots, etc). Missing values may be omitted if established to be MCAR or MAR.

Outliers

A value will be considered as an outlier if it can be justified as such both medically as well as statistically (e.g. via visual investigation via histograms and Q-Q plots as well as via Grubb's test for outliers).

Reference ranges for each liver marker can be found in Appendix D: Table 1.

Statistical software

Statistical analysis will be performed using R software version 3.5.3.

Reporting Conventions

When reporting the results of significance tests, precise p-values (e.g. p=0.034) will be reported rather than making exclusive reference to critical values. P-values ≥ 0.001 will be reported to 3 decimal places, p-values less than 0.001 will be reported at "<0.001".

References

(1) Banerjee R, Pavlides M, Tunnicliffe EM, Piechnik SK, Sarania N, Philips R, et al. Multiparametric magnetic resonance for the non-invasive diagnosis of liver disease. J Hepatol. 2014

(2) Blake L, Duarte RV, Cummins C. Decision analytic model of the diagnostic pathways for patients with suspected non-alcoholic fatty liver disease using non-invasive transient elastography and multiparametric magnetic resonance imaging. BMJ Open. 2016;6(9):e010507.

(3) Roeb E, Steffen M, Bantel H, Baumann U, Canbay A, Demir M, et al. [S2k Guideline non-alcoholic fatty liver disease.] Zeitschrift fur Gastroenterol. © Georg Thieme Verlag KG; 2015 5.

(4) Szczepaniak LS, Nurenberg P, Leonard D, Browning JD, Reingold JS, Grundy S, Hobbs HH, Dobbins RL. Magnetic resonance spectroscopy to measure hepatic triglyceride content: prevalence of hepatic steatosis in the general population. Am J Physiol Endocrinol Metab. 2005; 288(2): E462-8

Appendix A: RADIcAL 1 Study Objectives

Table 1: RADIcAL 1 Objectives and Outcome Measures/Endpoints

**Primary Objective**	**Primary Outcome Measures/Endpoints**
To investigate whether the introduction of Liver*MultiScan* as a standardised diagnostic test for liver disease can prove a cost-effective method in different EU territories.	Proportion of patients with suspected NAFLD incurring liver-related hospital consultations and/or liver biopsies, from the date of randomisation to end of study follow-up.
**Secondary Objective**	**Secondary Outcome Measures/Endpoints**
1/O. To investigate patient satisfaction with Liver*MultiScan* instead of existing care (with other liver investigations).	1/E. Patient feedback from patient satisfaction questionnaire, at baseline, and all follow-up visits to the end of the study.

2/O. To investigate certainty and frequency of diagnosis at points of time in the patient pathway.<br><br>3/O. To investigate which pathway was quicker to get to the diagnosis as recorded at final follow-up visit (including all corrections and additional investigations).<br><br>4/O. To measure what healthcare resources and costs were required in the two diagnostic pathways.<br><br>5/O. To investigate the cost-effectiveness of Liver*MultiScan* against standard of care.<br><br>6/O. To investigate skills/specialisation required.	2/E. Certainty of diagnosis is defined as a binary (yes/no vs. unlikely/probable) and frequency as (yes/probable vs. no/unlikely), at baseline and all follow-up visits to the end of the study.<br><br>3/E. Time from randomisation to diagnosis by the physician, as recorded at final follow-up visit.<br><br>4/E. Rates of liver-related outpatient investigations/consultations/hospital admissions per 400 patients during the study.<br><br>5/E. Cost of Liver*MultiScan* based on randomised comparison.<br><br>6/E. Personnel required to perform procedures and tasks from the date of randomisation to end of study follow-up.

Appendix B: Powering

Table 1: Confusion matrix for Transient Elastography (2, Table 1).

	+disease	-disease	sum
+test	51.156	9.888	61.044
-test	7.644	31.312	38.956
			100
Sensitivity		0.87	
Specificity		0.76	
Prevalence		58.8%	
Failure rate		18%	

Table 2: Confusion matrix for LMS (2, Table 1).

	+disease	-disease	sum
+test	50.568	2.884	53.452
-test	8.232	38.316	46.548
sum			100
Sensitivity		0.86	
Specificity		0.93	
Prevalence		58.8%	
Failure rate		5%	

Table 3: Biopsy reduction based on Transient Elastography and MpMRI sensitivity and specificity (2, Table 1).

**Fibroscan**			
Total	**1000**		**800**
Positive	498	*49.80%*	398
Negative	318	*31.80%*	254
Failures	184	*18.40%*	147
	**0.682**	**Total Bx %**	**0.682**
**LMS**			
Total	**1000**		**800**
Positive	508	*50.80%*	406
Negative	442	*44.20%*	354
Failures	50	*5.00%*	40
	**0.558**	**Total Bx %**	**0.558**

Appendix C: Timing of Outcome Measures

Table 1: Timings at which study data are collected

Timepoint	Control Arm	Study Arm
At Randomisation	Quality of Life Questionnaire & Resource use Questionnaire<br><br>Blood results: Standard liver markers mentioned in Analysis Method 3 and additional markers.	Quality of Life Questionnaire and Resource Use Questionnaire<br><br>Blood results: Standard liver markers mentioned in Analysis Method 3 and additional markers.<br><br>Patient Satisfaction Questionnaire following LMS
2 months	Quality of Life Questionnaire & Resource use Questionnaire	Quality of Life Questionnaire & Resource use Questionnaire
6 months	Quality of Life Questionnaire & Resource use Questionnaire	Quality of Life Questionnaire & Resource use Questionnaire
12 months	Quality of Life Questionnaire & Resource use Questionnaire	Quality of Life Questionnaire & Resource use Questionnaire

Appendix D: Reference Ranges

Table 1: Relevant blood markers are reference ranges.

Marker	Minimum	Maximum	Normal Range

Alkaline Phosphatase (IU/L)	10	1000	30-130
Gamma GT (IU/L)	10	2000	15-40
ALT (IU/L)	1	10000	10-45
AST (IU/L)	1	10000	15-42
Albumin (g/L)	1	100	32-50
Bilirubin (µmol/L)	0	1000	0-21
Total Serum Globulins (g/L)	0	300	20-35
IgG (g/L)	0	100	6.5-18.5
Haemoglobin (g/L)	0	250	120-175
WBCs (10^9/L)	0	300	4-11
Lymphocytes (10^9/L)	0	300	1-5
Neutrophils (10^9/L)	0	200	1.5-8
Platelets (10^9/L)	0	3000	150-450
Thioguanine Nucleotides (pmol/8x10^8 RBC)	0	1000	235-450
OGTT mmol/L	0		< 7.8
Fasting Blood Glucose mmol/l	0		3.9-5.4

Document Information

Document

Title	SAP_RADIcAL1_25Mar2020_V0.2
ID	b1739ee3-3c76-4913-bac1-6e3d3d9677f0
Status	Signed
Sender	Marika French (marika.french@perspectum.com)
Dept/business	Perspectum Diagnostics
Dept/business ID	perspectum-diagnostics3
PDF Certified	True

Signers

Name	Authentication
Matthias Dollinger (matthias.dollinger@klinikum-landshut.de)	Email
Dimitar Tonev (dimitar.tonev@yahoo.co.uk)	Email
Helena Thomaides-Brears (helena.thomaides-brears@perspectum.com)	Email
Marika French (marika.french@perspectum.com)	Email
Arina Kazimianec (arina.kazimianec@perspectum.com)	Email

History

Event	User	IP address	Date/Time
SAP_RADIcAL1_25Mar2020_V0.2 created by Marika French	Marika French (marika.french@perspectum.com)	62.255.31.130	Wed, 25 Mar 2020 14:01:37 +0000
SAP_RADIcAL1_25Mar2020_V0.2 emailed to Matthias Dollinger	Marika French (marika.french@perspectum.com)		Wed, 25 Mar 2020 14:01:40 +0000
SAP_RADIcAL1_25Mar2020_V0.2 emailed to Dimitar Tonev	Marika French (marika.french@perspectum.com)		Wed, 25 Mar 2020 14:01:40 +0000
SAP_RADIcAL1_25Mar2020_V0.2 emailed to Marika French	Marika French (marika.french@perspectum.com)		Wed, 25 Mar 2020 14:01:41 +0000
SAP_RADIcAL1_25Mar2020_V0.2 emailed to Arina Kazimianec	Marika French (marika.french@perspectum.com)		Wed, 25 Mar 2020 14:01:41 +0000
SAP_RADIcAL1_25Mar2020_V0.2 emailed to Helena Thomaides-Brears	Marika French (marika.french@perspectum.com)		Wed, 25 Mar 2020 14:01:42 +0000
SAP_RADIcAL1_25Mar2020_V0.2 visited by Arina Kazimianec <arina.kazimianec@perspectum.com>	Arina Kazimianec (arina.kazimianec@perspectum.com)	81.105.147.172	Wed, 25 Mar 2020 14:02:12 +0000
Browser geo-location provided: Lat 51.4474852, Long -0.9634334999999999	Arina Kazimianec (arina.kazimianec@perspectum.com)	81.105.147.172	Wed, 25 Mar 2020 14:02:16 +0000
SAP_RADIcAL1_25Mar2020_V0.2 visited by Marika French <marika.french@perspectum.com>	Marika French (marika.french@perspectum.com)	62.255.31.130	Wed, 25 Mar 2020 14:02:17 +0000
SAP_RADIcAL1_25Mar2020_V0.2 visited by Marika French <marika.french@perspectum.com>	Marika French (marika.french@perspectum.com)	62.255.31.130	Wed, 25 Mar 2020 14:02:37 +0000
SAP_RADIcAL1_25Mar2020_V0.2 visited by Matthias Dollinger <matthias.dollinger@klinikum-landshut.de>	Matthias Dollinger (matthias.dollinger@klinikum-landshut.de)	24.134.253.61	Wed, 25 Mar 2020 14:02:39 +0000
Signature applied (page 3)	Matthias Dollinger (matthias.dollinger@klinikum-landshut.de)	24.134.253.61	Wed, 25 Mar 2020 14:03:14 +0000
Signature applied (page 3)	Marika French (marika.french@perspectum.com)	62.255.31.130	Wed, 25 Mar 2020 14:03:21 +0000
Ticked checkbox for statement: I confirm that I am authorised to accept the terms of this document.	Matthias Dollinger (matthias.dollinger@klinikum-landshut.de)	24.134.253.61	Wed, 25 Mar 2020 14:03:35 +0000
Ticked checkbox for statement: I understand that accepting the terms of this document creates a legally binding obligation.	Matthias Dollinger (matthias.dollinger@klinikum-landshut.de)	24.134.253.61	Wed, 25 Mar 2020 14:03:35 +0000
Signer offered option to accept or reject document	Matthias Dollinger (matthias.dollinger@klinikum-landshut.de)	24.134.253.61	Wed, 25 Mar 2020 14:03:35 +0000
Clicked button with text: By clicking this button I hereby ACCEPT the terms of this document	Matthias Dollinger (matthias.dollinger@klinikum-landshut.de)	24.134.253.61	Wed, 25 Mar 2020 14:03:35 +0000

SAP_RADIcAL1_25Mar2020_V0.2 signed by Matthias Dollinger <matthias.dollinger@klinikum-landshut.de>	Matthias Dollinger (matthias.dollinger@klinikum-landshut.de)	24.134.253.61	Wed, 25 Mar 2020 14:03:35 +0000
Ticked checkbox for statement: I confirm that I am authorised to accept the terms of this document.	Marika French (marika.french@perspectum.com)	62.255.31.130	Wed, 25 Mar 2020 14:03:45 +0000
Ticked checkbox for statement: I understand that accepting the terms of this document creates a legally binding obligation.	Marika French (marika.french@perspectum.com)	62.255.31.130	Wed, 25 Mar 2020 14:03:45 +0000
Signer offered option to accept or reject document	Marika French (marika.french@perspectum.com)	62.255.31.130	Wed, 25 Mar 2020 14:03:45 +0000
Clicked button with text: By clicking this button I hereby ACCEPT the terms of this document	Marika French (marika.french@perspectum.com)	62.255.31.130	Wed, 25 Mar 2020 14:03:45 +0000
SAP_RADIcAL1_25Mar2020_V0.2 signed by Marika French <marika.french@perspectum.com>	Marika French (marika.french@perspectum.com)	62.255.31.130	Wed, 25 Mar 2020 14:03:46 +0000
Dimitar Tonev <dimitar.tonev@yahoo.co.uk> opened email for SAP_RADIcAL1_25Mar2020_V0.2	Dimitar Tonev (dimitar.tonev@yahoo.co.uk)	94.1.213.6	Wed, 25 Mar 2020 16:15:27 +0000
Auto reminder email sent to Dimitar Tonev <dimitar.tonev@yahoo.co.uk>	()		Thu, 26 Mar 2020 10:01:06 +0000
SAP_RADIcAL1_25Mar2020_V0.2 emailed to Dimitar Tonev <dimitar.tonev@yahoo.co.uk>	Dimitar Tonev (dimitar.tonev@yahoo.co.uk)		Thu, 26 Mar 2020 10:01:12 +0000
SAP_RADIcAL1_25Mar2020_V0.2 visited by Dimitar Tonev <dimitar.tonev@yahoo.co.uk>	Dimitar Tonev (dimitar.tonev@yahoo.co.uk)	94.1.213.6	Thu, 26 Mar 2020 10:06:37 +0000
Signature applied (page 3)	Dimitar Tonev (dimitar.tonev@yahoo.co.uk)	94.1.213.6	Thu, 26 Mar 2020 10:46:14 +0000
Ticked checkbox for statement: I confirm that I am authorised to accept the terms of this document.	Dimitar Tonev (dimitar.tonev@yahoo.co.uk)	94.1.213.6	Thu, 26 Mar 2020 10:46:53 +0000
Ticked checkbox for statement: I understand that accepting the terms of this document creates a legally binding obligation.	Dimitar Tonev (dimitar.tonev@yahoo.co.uk)	94.1.213.6	Thu, 26 Mar 2020 10:46:53 +0000
Signer offered option to accept or reject document	Dimitar Tonev (dimitar.tonev@yahoo.co.uk)	94.1.213.6	Thu, 26 Mar 2020 10:46:53 +0000
Clicked button with text: By clicking this button I hereby ACCEPT the terms of this document	Dimitar Tonev (dimitar.tonev@yahoo.co.uk)	94.1.213.6	Thu, 26 Mar 2020 10:46:53 +0000
SAP_RADIcAL1_25Mar2020_V0.2 signed by Dimitar Tonev <dimitar.tonev@yahoo.co.uk>	Dimitar Tonev (dimitar.tonev@yahoo.co.uk)	94.1.213.6	Thu, 26 Mar 2020 10:46:53 +0000
SAP_RADIcAL1_25Mar2020_V0.2 visited by Helena Thomaides-Brears <helena.thomaides-brears@perspectum.com>	Helena Thomaides-Brears (helena.thomaides-brears@perspectum.com)	62.255.31.130	Thu, 26 Mar 2020 13:15:13 +0000
Browser geo-location provided: Lat 51.7299563, Long -1.2087233000000002	Helena Thomaides-Brears (helena.thomaides-brears@perspectum.com)	62.255.31.130	Thu, 26 Mar 2020 13:15:21 +0000
Signature applied (page 3)	Helena Thomaides-Brears (helena.thomaides-brears@perspectum.com)	62.255.31.130	Thu, 26 Mar 2020 13:16:07 +0000
Signature applied (page 3)	Helena Thomaides-Brears (helena.thomaides-brears@perspectum.com)	62.255.31.130	Thu, 26 Mar 2020 13:16:57 +0000
Ticked checkbox for statement: I confirm that I am authorised to accept the terms of this document.	Helena Thomaides-Brears (helena.thomaides-brears@perspectum.com)	62.255.31.130	Thu, 26 Mar 2020 13:18:21 +0000
Ticked checkbox for statement: I understand that accepting the terms of this document creates a legally binding obligation.	Helena Thomaides-Brears (helena.thomaides-brears@perspectum.com)	62.255.31.130	Thu, 26 Mar 2020 13:18:21 +0000
Signer offered option to accept or reject document	Helena Thomaides-Brears (helena.thomaides-brears@perspectum.com)	62.255.31.130	Thu, 26 Mar 2020 13:18:21 +0000
Clicked button with text: By clicking this button I hereby ACCEPT the terms of this document	Helena Thomaides-Brears (helena.thomaides-brears@perspectum.com)	62.255.31.130	Thu, 26 Mar 2020 13:18:21 +0000

SAP_RADIcAL1_25Mar2020_V0.2 signed by Helena Thomaides-Brears <helena.thomaides-brears@perspectum.com>	Helena Thomaides-Brears (helena.thomaides-brears@perspectum.com)	62.255.31.130	Thu, 26 Mar 2020 13:18:21 +0000
SAP_RADIcAL1_25Mar2020_V0.2 emailed to Arina Kazimianec <arina.kazimianec@perspectum.com>	Arina Kazimianec (arina.kazimianec@perspectum.com)		Tue, 31 Mar 2020 10:24:48 +0000
SAP_RADIcAL1_25Mar2020_V0.2 visited by Arina Kazimianec <arina.kazimianec@perspectum.com>	Arina Kazimianec (arina.kazimianec@perspectum.com)	81.105.147.172	Tue, 31 Mar 2020 10:25:16 +0000
SAP_RADIcAL1_25Mar2020_V0.2 visited by Arina Kazimianec <arina.kazimianec@perspectum.com>	Arina Kazimianec (arina.kazimianec@perspectum.com)	81.105.147.172	Tue, 31 Mar 2020 10:25:29 +0000
Browser geo-location provided: Lat 51.4474728, Long -0.9634301	Arina Kazimianec (arina.kazimianec@perspectum.com)	81.105.147.172	Tue, 31 Mar 2020 10:25:34 +0000
Signature applied (page 3)	Arina Kazimianec (arina.kazimianec@perspectum.com)	81.105.147.172	Tue, 31 Mar 2020 10:48:49 +0000
Ticked checkbox for statement: I confirm that I am authorised to accept the terms of this document.	Arina Kazimianec (arina.kazimianec@perspectum.com)	81.105.147.172	Tue, 31 Mar 2020 10:49:26 +0000
Ticked checkbox for statement: I understand that accepting the terms of this document creates a legally binding obligation.	Arina Kazimianec (arina.kazimianec@perspectum.com)	81.105.147.172	Tue, 31 Mar 2020 10:49:26 +0000
Signer offered option to accept or reject document	Arina Kazimianec (arina.kazimianec@perspectum.com)	81.105.147.172	Tue, 31 Mar 2020 10:49:26 +0000
Clicked button with text: By clicking this button I hereby ACCEPT the terms of this document	Arina Kazimianec (arina.kazimianec@perspectum.com)	81.105.147.172	Tue, 31 Mar 2020 10:49:26 +0000
