# Supplementary material for: The Effect of Multi-Parametric Magnetic Resonance Imaging in Standard of Care for Nonalcoholic Fatty Liver Disease: Protocol for a Randomized Control Trial
Source: JMIR Res Protoc. 2020 Oct 26;9(10):e19189. doi: 10.2196/19189 (PMC7652684; doi:10.2196/19189)
Supplement: Multimedia Appendix 2 [file resprot_v9i10e19189_app2.pdf]

Ref. Ares(2016)669952 - 08/02/2016

Proposal Evaluation Form

EUROPEAN COMMISSION

Horizon 2020 - Research and Innovation Framework Programme

Evaluation Summary Report

Call: H2020-SMEINST-2-2015
Funding scheme: SME instrument phase 2
Proposal number: 719445
Proposal acronym: RADIcAL
Duration (months): 36
Proposal title: Non-invasive rapid assessment of chronic liver disease using Magnetic Resonance Imaging with LiverMultiScan
Activity: PHC-12-2015-P

N.	Proposer name	Country	Total Cost	%	Grant Requested	%
1	PERSPECTUM DIAGNOSTICS LTD	UK	3,460,486	100.00%	3,460,486	100.00%
	Total:		3,460,486		3,460,486	

Abstract:
Chronic liver diseases are called 'silent killers', as clinical symptoms only surface at late stages of the disease, when it is not treatable. Non-alcoholic fatty liver disease (NAFLD), a condition defined by fat accumulation in the liver, and its sub-type non-alcoholic steatohepatitis (NASH) are the major forms of liver disease. Untreated NAFLD/NASH can lead to cirrhosis and to liver failure. 450 million people worldwide suffer from fatty liver disease, which carries several health risks, and causes a huge socio-economic burden. At present, the only way of diagnosing and staging disease is with liver biopsy, which is costly, invasive, and carries some risk. Thus, clinicians are reluctant to use it for people suspected of having NAFLD/NASH. In addition, invasive biopsy is not optimal for serial assessment (e.g. monitoring transplant population for signs of liver rejection). Therefore, there is a lack of an accurate and non-invasive method of assessing liver disease and enabling treatment monitoring. Perspectum Diagnostics Ltd (PD) has developed LiverMultiScan, a novel, non-invasive, quantitative Magnetic Resonance Imaging software tool that displays high diagnostic accuracy for the early assessment of liver disease, and can be used for patient stratification. It has recently obtained regulatory clearance, but it requires additional clinical validation to assure its broad market acceptance and reimbursement. Thus, RADIcAL entails two clinical trials: a) a multi-centre randomised health economic study to validate the cost-effectiveness and added value of LiverMultiScan compared to the standard care pathway for chronic liver disease; and (b) a prospective clinical trial to demonstrate the high sensitivity and specificity of LiverMultiScan as a medical support tool for stratifying patients at high risk of liver transplant rejection.

Evaluation Summary Report

Evaluation Result

Total score: 14.18 (Threshold: 12)

Form information

Indicative Appraisal Scale per Sub-Criterion:
- Very Good to Excellent (4.5 – 5)
- Good to Very Good (3.5 – 4.49)
- Fair to Good (2.5 – 3.49)
- Insufficient to Fair (1.5 – 2.49)
- Insufficient (0-1.49)

Operational Capacity

Status: **Operational Capacity: Yes**

Criterion 1 - Impact

Score: **4.78** (Threshold: 4/5.00 , Weight: -)

Indicative Appraisal Scale per Sub-Criterion:
- Very Good to Excellent (4.5 – 5)
- Good to Very Good (3.5 – 4.49)
- Fair to Good (2.5 – 3.49)
- Insufficient to Fair (1.5 – 2.49)
- Insufficient (0-1.49)

Note: Where appropriate, the application should make reference to the successfully finalised Phase 1 project.
The proposal indicates in a convincing way that there will be demand/market (willing to pay) for the innovation when the product /solution is introduced into the market
Very Good to Excellent (4.5 – 5)

The targeted users or user groups of the final product/application, and their needs, are well described and the proposal provides a realistic description of why the identified groups will have an interest in using/buying the product/application, compared to current solutions available
Very Good to Excellent (4.5 – 5)

The proposal provides a realistic and relevant analysis of market conditions, total available market size and growth rate, competitors and competitive solutions and key stakeholders, clear opportunities for market introduction
Very Good to Excellent (4.5 – 5)

It is described in a realistic and relevant way how the innovation has the potential to boost the growth of the applying company
Very Good to Excellent (4.5 – 5)

The proposal demonstrates the alignment with the overall strategy of the participating SME(s) and the need for commercial and management experience, including understanding of the financial and organisational requirements for commercial exploitation as well as key third parties needed

Very Good to Excellent (4.5 – 5)

The innovation /solution has a clear European dimension both with respect to commercialisation and with respect to competitor / competition evaluation

Very Good to Excellent (4.5 – 5)

The strategy plan for commercialisation is described in a realistic and relevant way, including approximate time to market/deployment. Activities to be further developed after phase 2, including additional dissemination measures, are well outlined

Very Good to Excellent (4.5 – 5)

Measures to ensure "freedom to operate" (possibility of commercial exploitation) are realistic and there is a convincing strategy of knowledge protection, including current IPR filing status, IPR ownership and licensing issues. Regulatory and/or standard requirements are well addressed

Very Good to Excellent (4.5 – 5)

Overall perception including other pertinent factors (e.g. communication) not covered by the above questions (25% weight in the assessment)

Very Good to Excellent (4.5 – 5)

Criterion 2 - Excellence

Score: **4.70** (Threshold: 3/5.00 , Weight: -)

Indicative Appraisal Scale per Sub-Criterion:
- Very Good to Excellent (4.5 – 5)
- Good to Very Good (3.5 – 4.49)
- Fair to Good (2.5 – 3.49)
- Insufficient to Fair (1.5 – 2.49)
- Insufficient (0-1.49)

The innovation aims new market opportunities addressing EU/global challenges

Very Good to Excellent (4.5 – 5)

The included feasibility assessment demonstrates the technological/practical/economic viability of the innovation.

Very Good to Excellent (4.5 – 5)

The proposal provides a realistic description of the current stage of development (TRL 6 - see note 1- or similar for non-technological innovations) and added value of its innovation as well as very good understanding of the competing solutions. Includes good comparison with state-of-the-art, known commercial solutions, including costs, environmental benefits, gender dimension- see note 2-, ease-of-use and other features.
Note1: Technology Readiness level (TRL) is a measure used to assess the maturity of evolving technologies. Please see part G of the General Annexes.
Note 2: In relation to the project content, e.g. gender studies, clinical trials, etc.

Very Good to Excellent (4.5 – 5)

The objectives for the project as well as the approach and activities to be developed are consistent with the expected impact (commercialisation/deployment). Specifications for the outcome of the project and criteria for success are well defined

Very Good to Excellent (4.5 – 5)

The expected performances of the innovation are convincing and have the potential to be relevant from a commercial point of view (Value for money). It is potentially better than alternatives

Very Good to Excellent (4.5 – 5)

The proposal reflects a very good understanding of both risks and opportunities related to a successful market introduction of the innovation, both from a technical, commercial point of view.

Good to Very Good (3.5 – 4.49)

Overall perception including other pertinent factors not covered by the above questions (25% weight in the assessment)

Very Good to Excellent (4.5 – 5)

Criterion 3 - Quality and efficiency of implementation

Score: **4.70** (Threshold: 3/5.00 , Weight: -)

Indicative Appraisal Scale per Sub-Criterion:
- Very Good to Excellent (4.5 – 5)
- Good to Very Good (3.5 – 4.49)
- Fair to Good (2.5 – 3.49)
- Insufficient to Fair (1.5 – 2.49)
- Insufficient (0-1.49)

The proposal demonstrates that the project has the relevant resources (personnel, facilities, networks, etc.) to develop its activities in the most suitable conditions. If relevant, describes in a realistic way how key stakeholders / partners / subcontractors could be involved and why and how they were selected (subcontractors must be selected using best value-for-money principles). (Where relevant/) Participants in a consortium are complementary

Good to Very Good (3.5 – 4.49)

The team has relevant technical/scientific knowledge/management experience, and a very good understanding of the relevant market aspects for the particular innovation. If relevant the proposal includes a plan to acquire missing competences, namely

through partnerships or subcontracting (subcontractors must be selected using best value-for-money principles)
Good to Very Good (3.5 – 4.49)

Taking the project's ambition and objectives into account, the proposal includes a realistic time frame and a comprehensive implementation description
Very Good to Excellent (4.5 – 5)

The work package descriptions and major deliverables and milestones are realistic and relevant, including appropriateness of the allocation of tasks and resources, risk and innovation management
Very Good to Excellent (4.5 – 5)

Overall perception including other pertinent factors not covered by the above questions (25% weight in the assessment of this criterion)
Very Good to Excellent (4.5 – 5)

Subcontracting

Please note that, by default, the task is set to 'yes' even when the proposal does not foresee any subcontracting activities.
Subcontracting is acceptable in terms of "best value for money" : (only for NON-PHC-12-PROJECTS)

Task 1

Yes

Task 2

Yes

Task 3

Yes

Task 4

Yes

Task 5

Yes

Task 6

Yes

Task 7

Yes

Task 8

Yes

Task 9

Yes

Task 10

Yes

Scope of the proposal

Status: **Yes**

Comments:

Exceptional funding of third country participants/international organisations

A third country participant/international organisation not listed in General Annex A to the Main Work Programme may exceptionally receive funding if their participation is essential for carrying out the project (for instance due to outstanding expertise, access to unique know-how, access to research infrastructure, access to particular geographical environments, possibility to involve key partners in emerging markets, access to data, etc.). (For more information, see the Online Manual)

Based on the information provided in the proposal, we consider that the following participant(s)/international organisation(s) that requested funding should exceptionally be funded:
(Please list the Name and acronym of the applicant, Reasons for exceptional funding and the Requested grant amount.)

NA

Based on the information provided in the proposal, we consider that the following participant(s)/international organisation(s) that requested funding should NOT be funded:
(Please list the Name and acronym of the applicant, Reasons for exceptional funding and the Requested grant amount.)

NA

Use of human embryonic stem cells (hESC)

Does this proposal involve the use of hESC?

No

If yes, please state whether the use of hESC is, or is not, in your opinion, necessary to achieve the scientific objectives of the proposal and the reasons why. Alternatively, please also state if it cannot be assessed whether the use of hESC is necessary or not because of a lack of information.

Not provided
